# Reply to Comment: Evaluation of the Association of Omentin 1 rs2274907 A > T and rs2274908 G > A Gene Polymorphisms with Coronary Artery Disease in Indian Population: A Case–Control Study

**DOI:** 10.3390/jpm10040194

**Published:** 2020-10-26

**Authors:** Chandan K. Jha, Rashid Mir, Imadeldin Elfaki, Jamsheed Javid, Abdullatif Taha Babakr, Shaheena Banu, S. M. S. Chahal

**Affiliations:** 1 Department of Human Genetics, Punjabi University, Punjab 147002, India; chandujha58@gmail.com; 2 Prince Fahd Bin Sultan Research Chair, Department of Medical Lab Technology, Faculty of Applied Medical Sciences, University of Tabuk, Tabuk 71491, Saudi Arabia; rashidmirtabuk@gmail.com (R.M.); jamsheedjavidsjj@gmail.com (J.J.); 3 Department of Biochemistry, Faculty of Science, University of Tabuk, Tabuk 71491, Saudi Arabia; ielfaki@ut.edu.sa; 4 Department of Medical Biochemistry, Faculty of Medicine, Umm Al-Qura University, Makkah 57039, Saudi Arabia; abdullatiftaha@yahoo.com; 5 Sri Jayadeva Institute of Cardiovascular science and Research, Bangalore 560069, India; shanbanubanu@gmail.com

**Keywords:** Coronary Artery Disease (CAD), HWE, ARMS-PCR, Omentin 1

## Abstract

Coronary artery disease (CAD) is a major cause of death all over the world. CAD is caused by atherosclerosis which is induced by the interaction of genetic factors and environmental factors. Genome-wide association studies have revealed the association of certain gene polymorphisms with susceptibility to CAD. Omentin 1 is an adipokine secreted by the visceral adipose tissues and has been reported to have anti-inflammatory, cardioprotective, and enhances insulin sensitivity. In this study, we examined the role of omentin-1 common single nucleotide polymorphisms (SNPs) (rs2274907 A > T and rs2274908 G > A) in CAD. We conclude that the AT genotype and the T allele of the rs2274907 A > T is associated with Cad in the south Indian population. Our results indicated that the rs2274907 SNP may be associated with CAD in this population. This finding needs further validation in well-designed and large-sample size studies before being introduced in clinical settings.

Thank you very much for your interest in our study by Jha et al., 2019 [1]. We acknowledge that no genotyping method is 100% efficient and that genotyping errors can occur. However, we used the amplification refractory mutation system PCR (ARMS-PCR) and allele-specific PCR to genotype omentin 1 single nucleotide polymorphisms (SNPs) rs2274907 A > T and rs2274908 G > A. These methods are standard, fast, simple, and reliable for genotyping [2,3]. Results were repeated several times and once perfection was achieved, the remaining CAD samples were screened. Results were validated by different methods. Some CAD cases were screened by RFLP using restriction enzyme AccI (XmiI) or by Sanger sequencing. No difference in results from the ARMS-PCR was observed.

Our genotyping result deviated from HWE and this was probably due to many factors including population structure and/or sample size, purifying selection, copy number variation, inbreeding, or the substructure of this population [4,5]. The genotyping result was not very different from previous results in different populations (Table 1). Deviation from HWE was associated with loss or gain of heterozygosity [4]. In our study, it is likely that there was no loss or gain of the heterozygote genotype (AT) because the AT genotype percentage was 51% (Table 1). This percentage is more or less comparable to the AT genotype percentage obtained in some other populations (Table 1). We suggested that the deviation from HWE observed in our study occurred due to the substructure of this population. In support of this assumption is the recent study by Rathwa et al. [6] (Table 1), in which they report 0% AA genotype, 17.6% AT genotype, and more than 80% for the mutant genotype (TT). This indicates a complete shift to the mutant genotype (TT) in this population. Deviation from HWE observed in our study can also be due to the relatively small sample size used or other factors stated above [4,5]. We could not find the rs2274907 genotype distribution of an Indian population in other studies apart from the study by Rathwa et al. [6].

In Jha et al., 2019 [1], we report a potential association of the omentin SNP rs2274907 with CAD in a sample from a South Indian population. This result is consistent with previous studies conducted in Pakistani and Turkish populations [7,8], and to some extent in Iranian population [9]. Moreover, other studies reported an association of rs2274907 with type 2 diabetes [10,11], which is a risk factor for CAD.

In the database, the A allele is the major allele (https://www.ncbi.nlm.nih.gov/snp/rs2274907). Moreover, previous studies conducted in Pakistan and Iranian populations reported that the nucleotide change is from A to T [7,11,12]. The codon G**A**C is changed to G**T**C replacing the amino acid residue aspartate to valine at position 109 [13]. However, omentin 1 rs2274907 A > T is an SNP rather than a mutation that causes diseases [13].

In summary, we used a standard method for omentin SNPs genotyping. Our results indicated that the rs2274907 SNP may be associated with CAD in this population. This finding needs further validation in well-designed and large-sample size studies before being introduced in clinical settings.

## Figures and Tables

**Table 1:** Genotype distribution of omentin gene polymorphism rs2274907 in different populations.

Reference	AA	AT	TT	Population
1—Kohan et al., 2016 [12]	57.5	38.8	3.7	Iran
2—Khoshi et al., 2019 [11]	70.6	27.1	2.3	Iran
3—Rakowska, et al., 2017 [10]	48.50	41.92	6.59	Polish
4—Rathwa et al., 2019 [6]	0	17.6	82.4	Indian
5—Shazia Nazar et al., 2017 [7]	63	30	7	Pakistan
6—Ümit Yörük et al., 2014 [8]	6.66	48	45.33	Turkish
7—Zhang et al., 2015 [14]	9.8	43.5	46.7	Chinese
8—Jha et al., 2019 [1]	49	51	0	Indian
